# The Use and Abuse of Quinine

**Published:** 1882-01

**Authors:** 


### THE USE AND ABUSE OF QUININE.

We have recently reproduced, from some of our most valued exchanges, several articles protesting against the excessive use of quinine, and pointing out the dangers which attend its administration in enormous doses.

We deem the agitation of the subject both timely and appropriate, and, appreciating as we do the importance of the topic, we are glad that the question has been raised.

It is not surprising that a drug possessing the extensive applicability of quinine should be greatly abused. It is one of the few—the very few—specifics in our possession; and it is undoubtedly so useful in a large number of disorders and in a great variety of conditions, that there should be not infrequently an unjustifiable stretching of its application, and an unreasonable demand upon its therapeutic possibilities, is readily understood.

Nevertheless, its unwise and indiscriminate, as well as its excessive use should be condemned as not only unscientific, but as absolutely dangerous.

It has become the fashion to administer large doses of the drug in the height of a fever to reduce the pyrexia. The thermometer must determine the amount to be given —so many degrees call for so many grains. Then it must be exhibited as an anti-ferment. A hypothetical fermentation is raging in the blood, and the remedy must be heroically applied. It is also a valuable prophylactic, and consequently must be introduced in immense quantities to prepare the system for this or that ordeal—to anticipate a possible demand upon the vital forces, a possible strain upon an organ.

In the discussion of this question we do not desire to occupy an extreme position ; on the contrary, it is extreme practices that we are deprecating. Neither do we intend to discount the value of quinine, nor to wrest from it a single one of its many virtues ; but we do desire to repeat and to emphasize unmistakably the warning which has been sounded against the indiscriminate and excessive use—the abuse—of this powerful remedy, and to direct the attention of the medical profession to the danger which attends a heedless obedience to the edict of fashion, and an unquestioning reliance upon the example of authority.

